# Deep learning-based real-time detection of neurons in brain slices for in vitro physiology

**DOI:** 10.1038/s41598-021-85695-4

**Published:** 2021-03-16

**Authors:** Mighten C. Yip, Mercedes M. Gonzalez, Christopher R. Valenta, Matthew J. M. Rowan, Craig R. Forest

**Affiliations:** 1 Georgia Institute of Technology, George W. Woodruff School of Mechanical Engineering, Atlanta, 30332 USA; 2 Georgia Tech Research Institute, Atlanta, 30332 USA; 3 Department of Cell Biology, Emory University, Atlanta, 30322 USA

**Keywords:** Image processing, Machine learning, Patch clamp, Single-cell imaging

## Abstract

A common electrophysiology technique used in neuroscience is patch clamp: a method in which a glass pipette electrode facilitates single cell electrical recordings from neurons. Typically, patch clamp is done manually in which an electrophysiologist views a brain slice under a microscope, visually selects a neuron to patch, and moves the pipette into close proximity to the cell to break through and seal its membrane. While recent advances in the field of patch clamping have enabled partial automation, the task of detecting a healthy neuronal soma in acute brain tissue slices is still a critical step that is commonly done manually, often presenting challenges for novices in electrophysiology. To overcome this obstacle and progress towards full automation of patch clamp, we combined the differential interference microscopy optical technique with an object detection-based convolutional neural network (CNN) to detect healthy neurons in acute slice. Utilizing the YOLOv3 convolutional neural network architecture, we achieved a 98% reduction in training times to 18 min, compared to previously published attempts. We also compared networks trained on unaltered and enhanced images, achieving up to 77% and 72% mean average precision, respectively. This novel, deep learning-based method accomplishes automated neuronal detection in brain slice at 18 frames per second with a small data set of 1138 annotated neurons, rapid training time, and high precision. Lastly, we verified the health of the identified neurons with a patch clamp experiment where the average access resistance was 29.25 M$\Omega$ (n = 9). The addition of this technology during live-cell imaging for patch clamp experiments can not only improve manual patch clamping by reducing the neuroscience expertise required to select healthy cells, but also help achieve full automation of patch clamping by nominating cells without human assistance.

## Introduction

Whole-cell patch clamp electrophysiology, a gold standard technique in neuroscience, is a high-fidelity method used to monitor the biophysical mechanisms of neural activity at the single neuron level. Whole-cell patch clamp experiments allow the user to report current and voltage fluctuations at a spatiotemporal resolution beyond the capability of other techniques^1^. However, the technique is considered highly laborious and low throughput since it involves utilizing a glass micropipette to probe a cell individually—the trade-off for exceptional signal quality—preventing its widespread use for high-throughput screening. Typically, in vitro patch clamp experiments are done manually in which the user views an acute brain slice under a microscope, visually selects a neuron to patch, moves the pipette close to the cell, creates a high resistance (“giga-ohm”) seal between the pipette and cell, and breaks into the membrane to create a whole-cell configuration. These experiments allow scientists to monitor complex biophysical phenomena such as voltage and current fluctuations of single neurons.

One of the most crucial initial steps in the patch clamping process is identifying a healthy cell. The edges of a healthy neuron under DIC are often unclear and vary widely in shape and size. Moreover, the milieu of brain tissue not only consists of neurons, but also cerebrospinal fluid, blood vessels, and glia, among other extracellular content which induce significant light scattering under differential interference contrast (DIC), an optical technique widely used for observing unstained biological samples. While fluorescence microscopy may be used for identifying somas in acute slice patch clamp experiments, it is not always practical since it requires the use of dyes or genetically engineered production of fluorophores^2^. Rather, it is often desirable to image label-free, yet optically transparent samples which require the use of DIC.

Previous work has demonstrated success in automating cell detection in cultured cells^3,4^, via methods such as image segmentation and image enhancement techniques. Vicar et al. tested a handful of tools designed to detect cultured cells and compared them using the F1 score, a metric commonly used to measure object identification accuracy. The average F1 score reported for the methods which used DIC and additional preprocessing was 0.76. The average F1 score for the same methods on raw images was 0.50, indicating preprocessing may improve performance of object detection methods.

However, common image enhancement techniques, in concert with edge detection algorithms, are not robust enough for application in acute slice because the nature of the images under DIC yields more scattering than cultured cells. In addition, there are several cell segmentation and tracking methods that are not directly applicable to cell detection under DIC in tissue^5^. To overcome this obstacle, this work adapts a deep neural network to identify neurons in acute slice—particularly neurons in the layer 5 cortex of the mouse brain. While there has been a myriad of convolutional neural networks (CNNs) used for identifying cells, most applications are used on images post-experiment either for cell detection on slides, cell cultures, or for cell segmentation of 3-D connectomics^6–10^.

Since this is such a critical task, often requiring significant experience to identify healthy cells, automation of the cell identification and selection process is a difficult, necessary step towards completing full automation of patch clamp as well as in assisting novices how to identify cells. Research groups enabling the automation of patch clamp have alluded to the potential benefits of automating this task, though the problem is not yet fully resolved^11–13^. Koos et al. have recently shown a CNN that identifies somas under DIC, though their network required substantial time and over 31,000 annotated neurons for training^14^. In this study, we aimed to achieve similar accuracy on a smaller, faster CNN that can quickly nominate cells for patch clamp experiments. Our deep learning-based method, quantified by F1 scores and mean average precision (mAP), is comparable to published work on cultured cell identification and other deep learning based solutions for cell detection. Thus, we show that transfer learning using the YOLOv3-CNN architecture can require minimal training resources and enable fast, accurate neuronal detection for images gathered on live, acute brain slices.

## Methods

**Figure 1:**
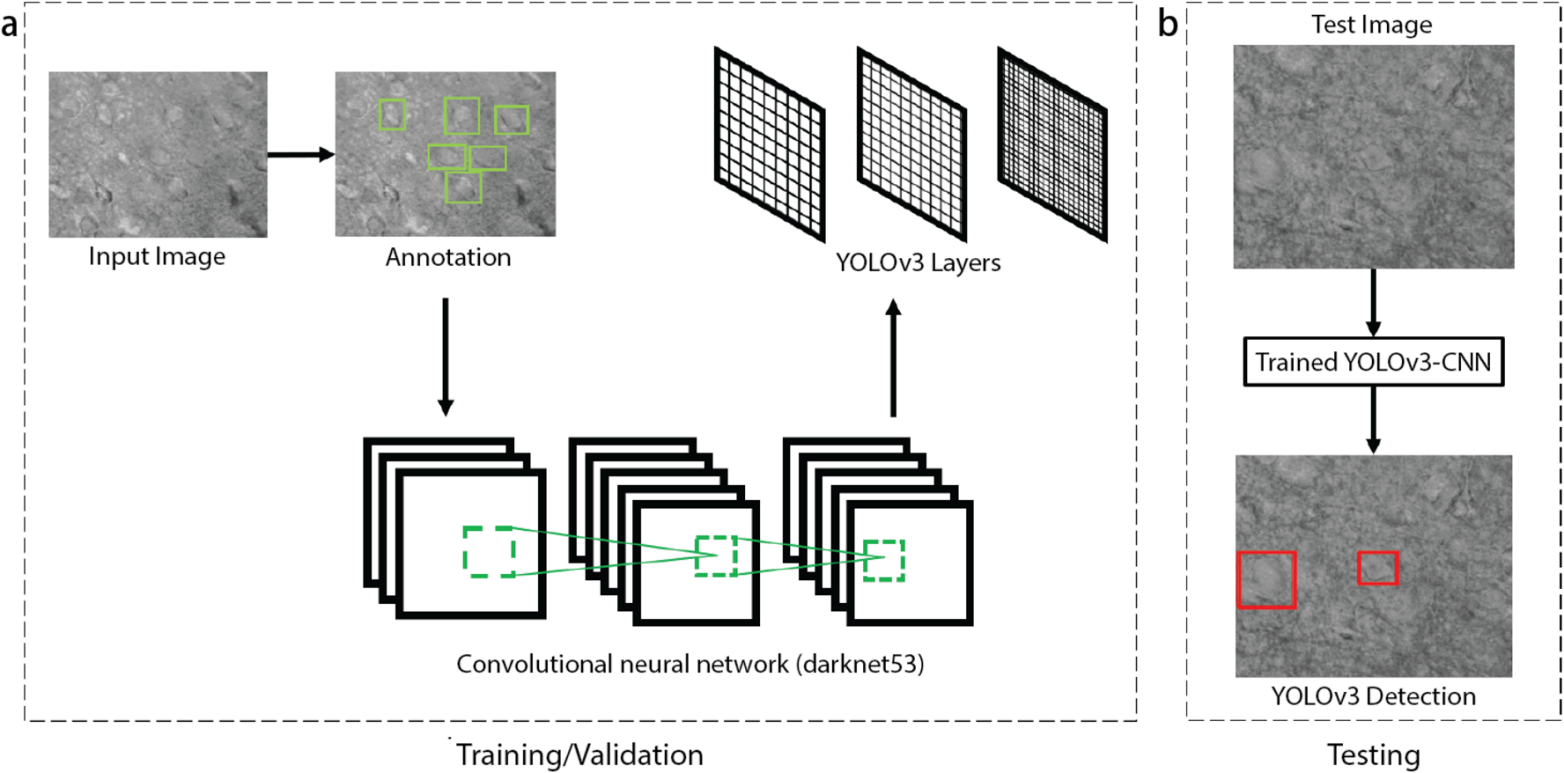
After initial (**a**) training and validation using annotated input images, testing (**b**) shows a successful detection of neurons in unannotated, unaltered images.

For the purposes of automated neuronal detection in acute brain slices, we utilize the default architecture of the YOLOv3 neural network, most notable for its speed and accuracy of detection^15,16^. In order to increase speed in object detection, YOLO reframes object detection as a single regression problem, mapping straight from image pixels to bounding box coordinates and class probabilities. In addition, YOLO looks globally on the entire image when making predictions. The primary motivation for selecting an architecture optimized for speed and accuracy is to apply the network to a video or real-time imaging. Thus, our methods include using transfer learning with the YOLOv3 architecture to provide a default model to fine-tune. A representative workflow is represented in Fig. 1.

### Implementation

#### Acquisition of acute brain slice images

All acute brain slice samples and images were captured utilizing the hardware and software configuration according to Kolb et al.^11^. The system was based on a conventional electrophysiology setup (SliceScope Pro 3000, Scientifica Ltd), and the samples were imaged using a 40$\times$ objective (LUMPFLFL40XW/IR, NA 0.8, Olympus) on a motorized focus drive, illuminated under DIC with an infrared light-emitting diode (Scientfica), and captured with a Rolera Bolt camera (QImaging). All animal procedures were in accordance with the US National Institutes of Health Guide for the Care and Use of Laboratory Animals and were approved by the Institutional Animal Care and Use Committee at the Georgia Institute of Technology.

#### Annotation procedure

Annotations were made manually using LabelImg, an open source graphical image annotation tool written in Python^17^. Annotations were saved as XML files in PASCAL VOC format, the format as used by ImageNet^18^. Healthy neurons were annotated and labeled by drawing a bounding box around them. The rectangular boxes varied in size and were allowed to intersect with each other.

The training, validation, and test data sets consisted of 1280 $\times$ 1024, 8-bit raw images of acute slices under DIC. Within the training and validation data sets, 369 original, raw images were used with a total of 1138 annotated neurons. For the trained CNN test data set, a smaller set of 37 images was used containing 107 annotated neurons. Since we are using transfer learning on a pre-trained model, a smaller data set for training and validation is appropriate to obtain sufficient accuracy. All data sets will be made publicly available at autopatcher.org.

#### Convolutional neural network: YOLOv3

As mentioned previously, healthy cells from raw images of acute slice under DIC are difficult even for humans to identify. As an immediate effort to improve our ability to detect cells, using Python, we applied traditional image enhancement techniques to increase contrast and sharpen edges to the human eye. However, the advantages of preprocessing training data in machine learning have not been confirmed, motivating this work to also compare two training models to reveal whether or not image enhancement (using custom Python scripts and the OpenCV library) improved the performance of the neural network. Histogram equalization, an image processing technique commonly used for improving contrast, both enhances contrast and preserves detail in the images. We compared a network trained on raw, unaltered images to a network trained on histogram equalized images. Histogram equalization was the only image enhancement technique used to create training and validation data sets, so hereafter we will refer to those data sets as “enhanced” for conciseness. Data sets without image enhancement will be referred to as “unaltered.”

For both the unaltered image and the enhanced image data sets, they were randomly divided into a training and validation set at a 10:1 ratio. The input image resolution was set to 416 $\times$ 416 pixels. While downsampling the images to 416 $\times$ 416 introduces some unintended artifacts of reduced resolution, there is a desired trade-off between computational time and accuracy. Secondly, the dimensions of the input image are resized while maintaining the image aspect ratio. For example, the longer dimension, 1280, is scaled to 416 and the secondary dimension, 1024, is scaled to 332.8 pixels. The remaining pixel area is black pixels. The YOLOv3 network architecture consists of a backbone network called Darknet-53, an up-sampling network, and the detection layers called YOLO layers^15,16^.

As stated previously, transfer learning fine-tunes a pre-trained neural network model. Since the model does not need to be trained from scratch, transfer learning is often a suitable choice when training with limited training and validation data sets. Moreover, transfer learning has the potential to improve network performance and training time^19^. The initial model of our CNN was pre-trained on the Imagenet data set^16^. The final 3 layers were initially trained with our custom acute brain slice data set for 10 epochs before all layers were unfrozen and the entire network was trained on the data set for 40 more epochs for a total of 50 epochs. If loss reached a steady state value, the training would stop early.

In addition, YOLOv3 object detection utilizes non-max suppression (NMS) which was also utilized here to classify the determined predictions. The intersection over union for NMS was set to 0.45 as based on machine learning standards.

Training computations were conducted on a desktop PC with a 3.7 GHz Intel Core i7-8700K, 32GB RAM memory and an NVIDIA GeForce GTX 1080. For trained model evaluations, the software was run on a notebook PC with a 2.8 GHz Intel Core i7-7700HQ and 16GB RAM memory.

### Evaluation metrics of YOLOv3 performance

Generally, trained networks can be assessed quantitatively through the metrics of precision (P) and recall (R),

1 $$\begin{aligned} P = \frac{T_p}{T_p + F_p}, \qquad R = \frac{T_p}{T_p + F_n}, \end{aligned}$$

where $T_{p}$ is the number of true positive classifications, $F_{n}$ is the number of false negatives, and $F_{p}$ is the number of false positives. Precision represents how likely a prediction will be accurate. On the other hand, recall represents how accurate the model is based on correct classifications and classifications it failed to identify. Therefore, for an ideal model, it is desired to have both precision and recall equal to 1, or 100%.

In this study, PASCAL VOC-style Average Precision (AP) at a single intersection over union (IOU) threshold of 0.45 was calculated to evaluate the models trained by the loss function of YOLOv3^20^. Although the general definition of AP is the area under the precision-recall curve,

2 $$\begin{aligned} AP = \int _{0}^{1} P(R) dR, \end{aligned}$$

the interpolated precision-recall, “P(R)”, curve is piece-wise constant. Therefore, with the number of recall values, we define AP as described in Cartucho et al.^21^.

Since AP is the integration of precision with respect to recall, and the ideal precision and recall values are both 1, the ideal AP is also 1, or 100%. Mean average precision (mAP) is especially helpful for multi-class studies, since it is the average AP of each class the network can identify. While there is only one class (‘neuron’) in this study, we will use the common notation of mAP hereafter.

Another common metric used for quantifying the performance of neural networks is the F1 score^10^,

3 $$\begin{aligned} F1 = 2\frac{PR}{P+R}, \end{aligned}$$

which is particularly useful when determining the optimal balance between precision and recall. Since the ideal network would yield precision and recall equal to 1, the F1 score would then also be 1.

The last metric used describes the accuracy of the model using true positives (TP), false positives (FP), and the ground truth (GT) annotations where,

4 $$\begin{aligned} \text {Accuracy} = \frac{TP}{GT+FP}, \end{aligned}$$

such that the ideal model would be 100% accurate should all its guesses match the ground truth annotations without false positives.

### Real-time detection and patch clamp validation

#### Brain slice preparation

All animal procedures were in accordance with the US National Institutes of Health Guide for the Care and Use of Laboratory Animals and were approved by the Institutional Animal Care and Use Committee at the Georgia Institute of Technology. For the brain slice experiments, male mice (C57BL/6, P31–P46, Charles River) were anesthetized with isofluorane, and the brain was quickly removed. Coronal sections (300 m thick) were then sliced on a vibratome (Leica Biosystems VT1200S) while the brain was submerged in ice-cold sucrose solution containing (in mM) 40 NaCl, 4 KCl, 1.25 $\hbox {NaH}_{2}\hbox {PO}_{4}\cdot \hbox {H}_{2}\hbox {O}$, 7 MgCl$_{2}$, 25 NaHCO$_{3}$, 10 D-Gluocse, 0.5 CaCl$_{2}\cdot 2\hbox {H}_{2}$O, 150 Sucrose (pH 7.3–7.4, 300–310 mOsm). The slices were incubated at $37{}^{\circ }\hbox {C}$ for 1 h in neuronal artificial cerebro-spinal fluid (ACSF) consisting of (in mM) 124 NaCl, 2.5 KCl, 1.25 NaH$_{2}$PO$_{4}\cdot \hbox {H}_{2}$O, 1.3 MgCl$_{2}$, 26 NaHCO$_{3}$, 10 D-Gluocse, 2 CaCl$_{2}\cdot 2\hbox {H}_{2}$O, 1 L-Ascorbate $\cdot$ H$_{2}$O (pH 7.3–7.4, 290–300 mOsm). Prior to recording, the slices were maintained at room temperature for at least 15min (22–25 ${}^{\circ }\hbox {C})$. The sucrose solution and neuronal ACSF were bubbled with 95% O2/5% CO2. Recordings were performed in mouse primary visual area.

#### Patch-clamp recording

Borosilicate pipettes were pulled on the day of the experiment using a horizontal puller (P-97, Sutter Instruments) to a resistance of 4–5 M$\Omega$. The intracellular solution was composed of (in mM) 135 K-Gluconate, 10 HEPES, 4 KCl, 1 EGTA, 0.3 Na-GTP, 4 Mg-ATP, 10 Na_2_-phosphocreatine (pH 7.2–7.3, 290–300 mOsm). Recordings were performed at room temperature with constant superfusion of oxygenated neuronal ACSF. During the patch clamp experiment, the YOLOv3 neuron detection algorithm, using the unaltered trained network, was run on the desktop PC with the NVIDIA GeForce GTX 1080 GPU on a custom python script to interact with the Rolera Bolt camera. Pipette pressure during patch clamp steps was digitally controlled and pipettes were cleaned according to Kolb et al.^11,22^.

## Results

### YOLOv3 neuron detection

**Figure 2:**
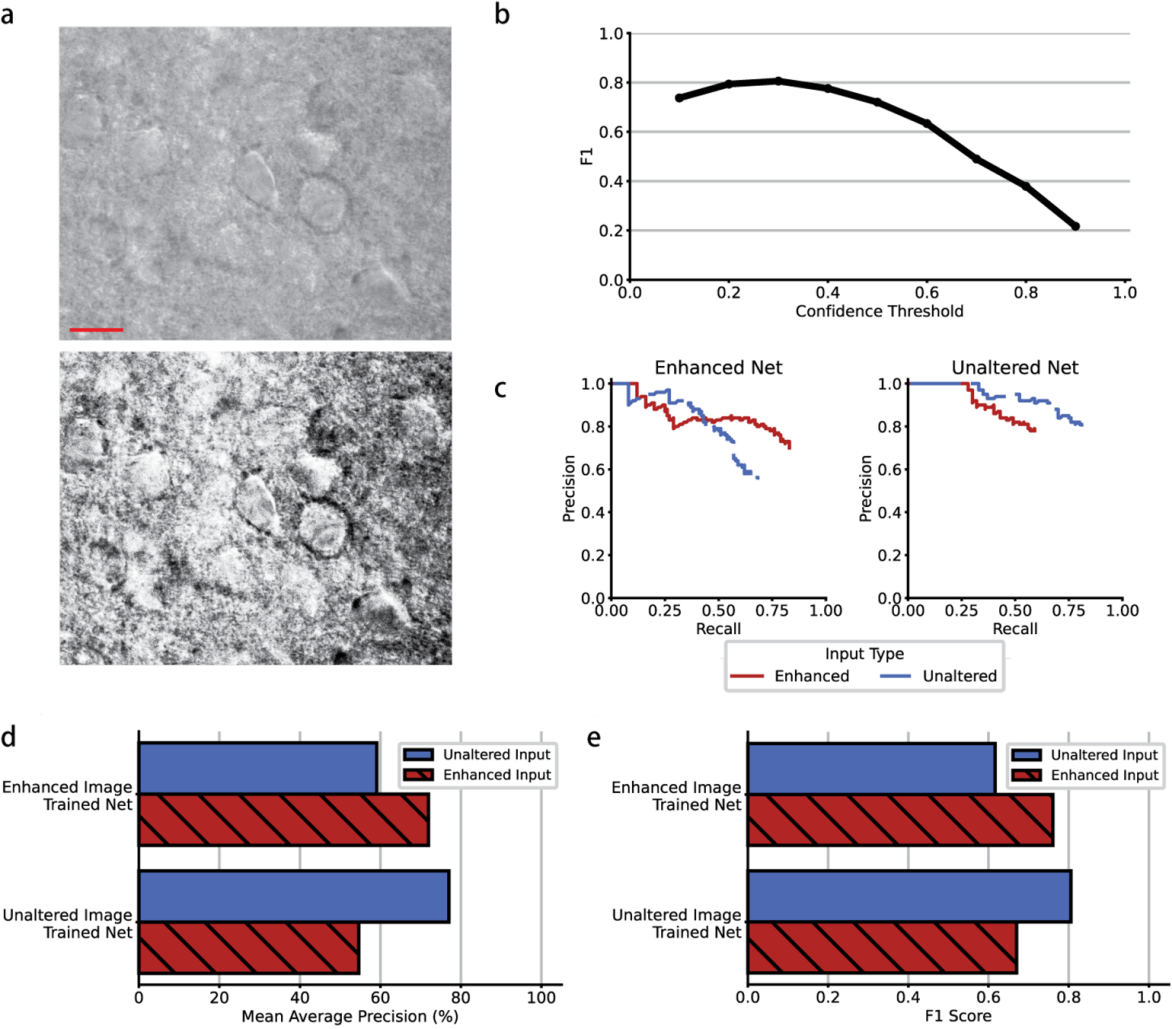
(**a**) Representative example of unaltered (top) and enhanced (bottom) images of acute slice under DIC. (**b**) Representative plot of F1 score vs confidence threshold, demonstrating peak in F1 score at a confidence threshold of 0.3. (**c**) *left* Relationship between precision and recall for the enhanced network tested on enhanced and unaltered data set test images. *right* Relationship between precision and recall for the unaltered network tested on enhanced and unaltered data set test images. (**d**) Summary of mean average precision of both networks for both enhanced and unaltered inputs. (**e**) Summary of F1 score of unaltered and enhanced networks for both enhanced and unaltered inputs. $\hbox {Scale bar} = 10\,\upmu$m.

As previously described, we compared the performance of a network trained on only unaltered images to a network trained on enhanced images. A representative example image of an unaltered image and an enhanced image is shown in Fig. 2a.

When evaluating the performance of these networks, two metrics can be adjusted to increase or decrease the accuracy of the network’s predictions: (1) the confidence threshold, a measure of the probability that a prediction contains an object and (2) the aforementioned IOU. In order to optimize confidence threshold, we evaluated the networks with the F1 scores, using a constant IOU according to machine learning standards (0.45) and a range of confidence thresholds from 0.1 to 0.9. The relationship between F1 score and confidence threshold for the unaltered network tested on unaltered images can be found in Fig. 2b. Since there is a peak in F1 score over the range of confidence thresholds, the optimal confidence threshold of 0.3 was used for all further analyses.

In order to test the unaltered and enhanced trained YOLOv3 networks, we evaluated and compared their performance using precision recall and mAP. Precision recall is a useful measure of prediction success. In information reasoning, precision measures the accuracy or percentage of correct predictions, while recall measures how good it is at finding all the correct objects. Figure 2c shows the precision-recall curves for both networks, tested on both enhanced and unaltered inputs. The mAP is the area under this curve, summarized in Table 1 and displayed in Fig. 2d.

The unaltered trained network was trained on images without preprocessing. The mean average precision of this network tested on an unaltered test image data set was 77.00%, while the same network tested on a enhanced data set was 54.62%. Conversely, the enhanced trained network was trained on images enhanced with histogram equalization^23^. The mean average precision of this network was 59.10% with unaltered test images and 71.93% on enhanced images.

The F1 scores of both networks are summarized in Table 2 and displayed in Fig. 2e. The F1 scores of the unaltered network tested on unaltered and enhanced images were 0.8 and 0.67, respectively. The F1 scores for the enhanced network were 0.61 and 0.76, respectively.

**Table 1:** Mean average precision of unaltered and enhanced trained networks tested on unaltered and enhanced input images.

	Unaltered input (%)	Enhanced input (%)
Unaltered trained network	77.00	54.62
Enhanced trained network	59.10	71.93

**Table 2:** F1 score of unaltered and enhanced trained networks tested on unaltered and enhanced input images.

	Unaltered input	Enhanced input
Unaltered trained network	0.80	0.67
Enhanced trained network	0.61	0.76

#### Inference results

**Figure 3:**
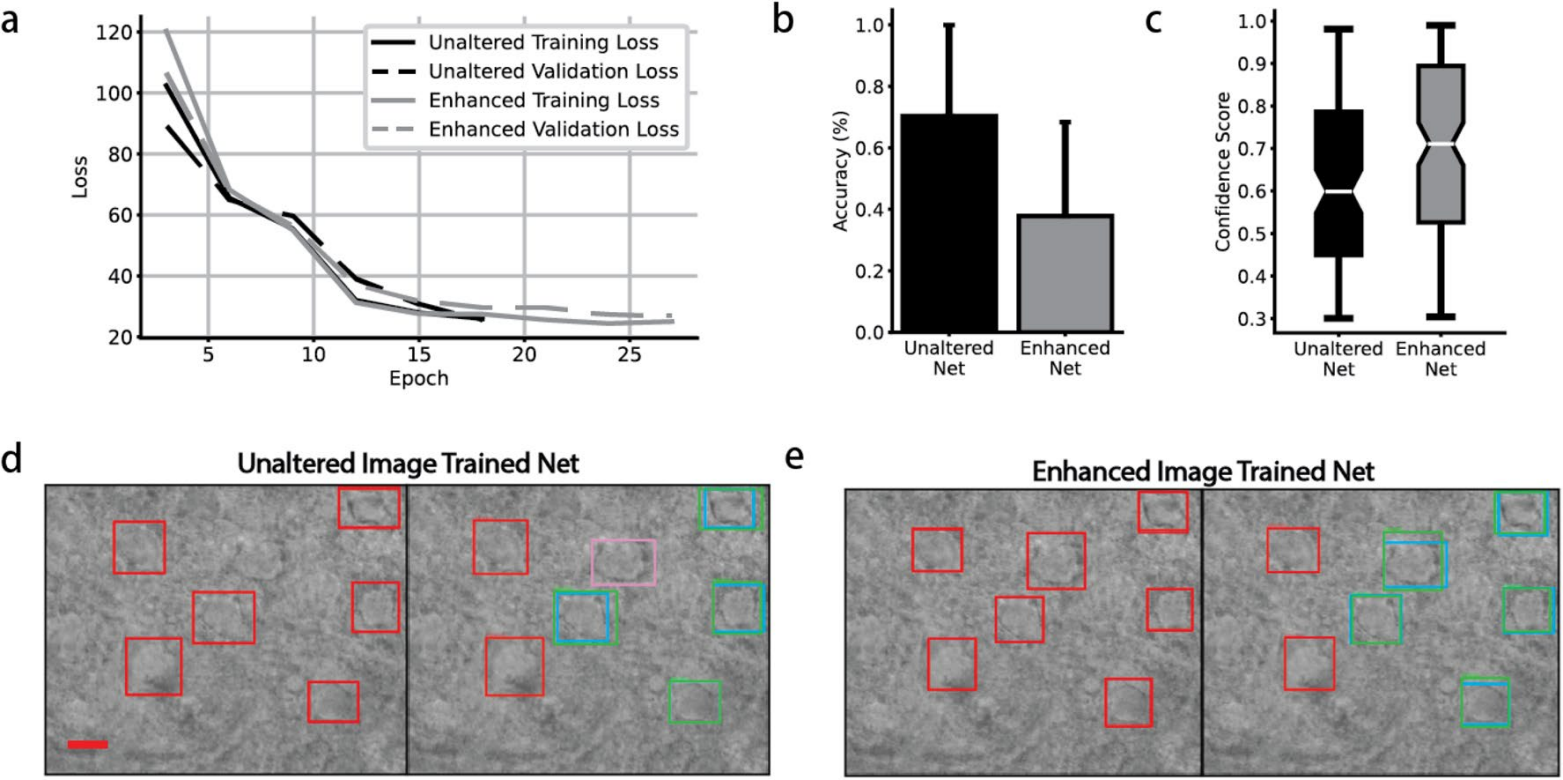
(**a**) Convergence on training and validation loss with respect to number of epochs. Black lines represent the unaltered trained model losses, and gray represents the enhanced trained model losses. Solid lines represent training loss, and dashed lines represent validation loss. (**b**) The bar chart shows mean ± SD comparison of the average accuracy between the unaltered net and enhanced net on the unaltered data set test images. A student’s t-test ($\alpha$ = 0.05) acknowledges that the difference between the means is statistically significant; t(36)=5.12, p$<0.001$. (**c**) Box plot comparison of the confidence scores distribution for unaltered and enhanced networks tested on the unaltered data set test images. The notches represent the confidence interval around the median using a Gaussian-based asymptotic approximation. The ends of the boxes are at the first and third quartiles while the whiskers represent the minimum and maximum confidence scores. (**d**,**e**) Example of both networks identifying neurons in a test image. *left* initial prediction (red) of neurons. *right* bounding boxes for annotation (blue), correct prediction (true positive—green), incorrect prediction (false positive—red), and undetected neurons (false negative—pink). Scale bar: $10 \upmu$m.

The training loss and validation loss for both networks shown in Fig. 3a highlight the neural network quickly fitting to the training set and converging towards a steady-state of trained weights.

While the results of the models over enhanced images provide relevant information over the precision of the networks, generally, preprocessing each frame during a real-time live-imaging experiment would cause latency issues so further accuracy and confidence score distributions are studied using only the unaltered test images data set. As seen in Fig. 3b, the mean accuracy of the unaltered net was $0.703\, \pm \,$0.296 while the enhanced net was $0.378\, \pm \,$0.306, student’s t-test p $<\,0.001$. Figure 3c shows the confidence scores distribution for unaltered and enhanced networks tested on the unaltered data set test images. The notches represent the confidence interval around the median, 0.599 and 0.711, respectively. The ends of the boxes are at the first and third quartiles while the whiskers represent the minimum and maximum confidence scores.

Examples of each network identifying neurons in a test image can be found in Fig. 3d,e. On the left half side of each subfigure display, each model’s reasoning for a neuron is overlaid with red prediction bound boxes on a representative test image. On the right half side of each subfigure, the prediction of the CNN is graded against the expert-annotated test image. A green box represents a correct prediction (true positive). A blue box represents the original annotated bounding box. A red box represents a model prediction that is a false positive. And lastly, a pink box denotes an annotated neuron that was missed by the model (false negative).

Although our average reported inference time for an image was $580 \pm 147$ ms, this can be attributed to testing the trained models on the CPU of the notebook PC described in the “Methods” section. Average inference time testing the trained models on the GPU described in the “Methods” section was $56.7 \pm 1.43$ ms. This provides an 18 frame per second real-time detection rate (Supplementary Video 1). Furthermore, the training time for each of the models was 18 minutes.

#### Patch clamp experiments

**Figure 4:**
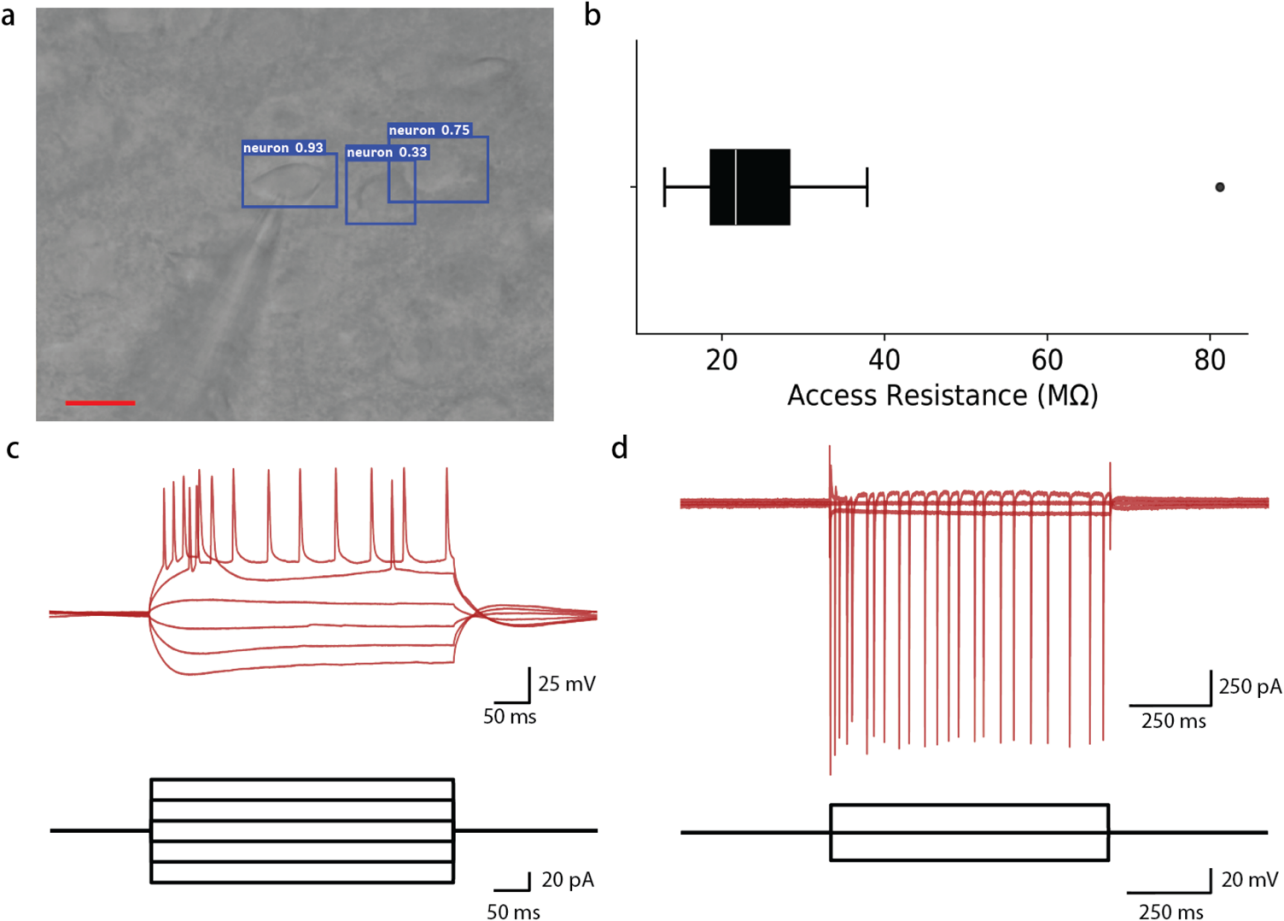
(**a**) Image of a network-identified neuron in patch clamp whole-cell configuration. The blue bounding boxes indicate identified neurons. The numbers ranging from 0 to 1 indicate the network’s confidence that the box contains a neuron. The pipette recording electrode is visible on the lower left quadrant resting on the leftmost of the three identified neurons. (**b**) Distribution of access resistance indicate that 8 out of 9 cells (89%) yielded high quality whole cell recordings. The white line indicates the median (21.7), the box width indicates the interquartile range (9.6), and the whiskers indicate the range of the data, excluding outliers. (**c**) Representative current clamp trace and (**d**) voltage clamp trace from a neural network-identified neuron in whole-cell configuration. Scale bar: $10 \,\upmu$m.

To validate the health of the identified cells, we performed a set of patch clamp experiments on neurons identified by one of the trained neural networks. We chose to use the unaltered trained network since it demonstrated the greatest mAP and F1 scores. A representative image of a neuron identified by the network in patch clamp whole-cell configuration is shown in Fig. 4a. The distribution of access resistances from these experiments (n = 9) is displayed as a box plot in Fig. 4b. The average access resistance was 29.25 M$\Omega$. The ends of the boxes are the first and third quartiles (18.7 M$\Omega$ and 28.27 M$\Omega$, respectively) while the whiskers are located at 12.97 M$\Omega$ and 37.86 M$\Omega$. Further, 8 of 9 patched cells were within the accepted range among patch clamp experts $(< 40 \,\hbox {M}\Omega )$^11^. Representative current clamp and voltage clamp traces are shown in Fig. 4c,d, respectively.

## Discussion and conclusion

The use of patch clamping in mammalian brain slices is well documented^1,24^, yet the majority of the technique is still done manually. While research groups have made improvements to automate many of the painstaking steps involved with patch clamping in vitro^11,12,22,25^, the initial act of selecting a healthy neuron to patch still has yet to be resolved.

In this study, we developed a method for detecting neurons in acute, rodent brain slice for anticipatory use towards assisting patch clamp experiments. We then validated the method’s ability to identify healthy cells by patch clamping neurons identified by the best performing network (unaltered trained network). The application of this neural network in the context of patch clamp has great potential to help fully close the loop towards complete automation of the patch clamp technique on acute brain slices and reduce the need for immense training and skills required for manual identification of healthy cells. The YOLO network architecture’s speed and accuracy are conducive for nominating healthy neurons in acute brain slice in real-time with a display and detection rate of 18 frames per second. Thus, this neuron detection method is a tool not only valuable for initially identifying neurons for patching, but it could also provide tracking of the cell location as the slice is moved during an experiment to aid in throughput and quality of the recording. In addition, this work could have several secondary benefits that address the requirements for highly reproducible data^26^. By removing the user from the cell selection process, it inherently reduces experimenter bias, reduces type I & II error and increases experimental rigor.

Both networks performed best when tested with input images that were similar to their respective training data sets. Interestingly, while preprocessing may have improved the contrast of cell boundaries to the human eye, it did not improve the network performance most likely because the enhanced contrast has introduced artifacts that interfere with the boundaries of the cells. The statistically significant difference in the mean accuracy between the unaltered and enhanced networks support this finding by a student’s t-test ($\alpha$ = 0.05), p $< 0.001$. Most likely, the non-linear contrast enhancement degraded the image and reduced detectable linear features that may improve a model’s precision and accuracy. Furthermore, while the median for the enhanced net is higher than the unaltered net in the distribution of confidence scores, the lower mean accuracy score for the enhanced net shows it may be misleading to determine a neural network’s efficiency and precision based on its confidence score.

This study also had some limitations. Since only one class of neurons were chosen, and image acquisition was time-consuming, there was a limited number of images—thus, neurons—for training the neural network for neuron detection. However, further collaboration with patch clamp research groups can help increase the speed and quality of image acquisition. While Koos et al. have conducted a similar deep learning-based method for neuron detection in slice^14^, our method achieves similar precision while being more efficient and user-friendly. Using YOLOv3, our F1 score of 80% is comparable to the F1 score of 83.5% by Koos et al. In addition, our CNN has a reduced neural net training time of 0.3 h while Koos et al. took 159 h—a 98% reduction. Thus, the deep learning method presented here demonstrates the promise of implementing CNNs even further in the field of electrophysiology. Our study introduces the feasibility of performing classification tasks on acute brain slices by using a sparsely annotated data set (our sparsely annotated data set of 1138 neurons compared to 6344 annotated neurons by Koos et al.) Furthermore, we have demonstrated the advantage of transfer learning in improving network performance, especially when limited data is available, and confirmed that current image enhancement techniques do not necessarily help neural network performance.

The image enhancement techniques used in this method are not comprehensive, and other image enhancement techniques can also be explored particularly for low-contrast, gray-scale images^27^ and use of Kalman filtering^28^. In addition, future work can include customizing the YOLO architecture to optimize network training for our data sets, training on a greater number of annotated images, and upgrading the object detection architecture to YOLOv4 for improved precision. Pruning the YOLO architecture can also improve neuron detection speed^29^. Moreover, this technique could be used to detect and analyze subcellular features such as spines, dendrites, or axons.

Current software will be publicly available on Github (https://github.com/mightenyip/neuronDetection). Future work will focus on applying existing models to detecting neurons in real-time prior to patch clamp experiments. In addition, data augmentation methods and the detection model will be optimized to further improve the detection accuracy. Moreover, additional classes can be annotated to expand neuronal detection to other types of neurons. Thus, paving the future for an object detection-based neural network capable of reasoning the entire environment of an acute brain slice.

## Supplementary information

Supplementary Information.
